# Experimental evaluation of a hybrid evaporative and groundwater cooling system for enhancing photovoltaic efficiency in arid climates

**DOI:** 10.1038/s41598-025-22704-w

**Published:** 2025-10-13

**Authors:** Deyaa M. N. Mahmood, Issam M. Ali Aljubury, Najah M. L. Al Maimuri, Mudhar A. Al-Obaidi, Farhan Lafta Rashid, Arman Ameen, Shaohua Dong, Yasir Mukhtar

**Affiliations:** 1 Technical Instructor Training Institute, Middle Technical University, Baghdad, 10074 Iraq; 2 College of Engineering, University of Baghdad, Baghdad, Iraq; 3 Building and Construction Techniques Engineering Department, College of Engineering and Engineering Techniques, Al-Mustaqbal University, Hillah, Babylon 51001 Iraq; 4 Petroleum Engineering Department, College of Engineering, University of Kerbala, Karbala, 56001 Iraq; 5 Department of Building Engineering, Energy Systems and Sustainability Science, University of Gävle, 801 76 Gävle, Sweden; 6 Pipeline Technology and Safety Research Center, China University of Petroleum-Beijing, Beijing, 102249 China; 7 Faculty of Engineering and Technology, Nile Valley University, Atbara, Sudan; 8 Innovation Center, MB University Belgrade, Teodora Drajzera 27, Belgrade, Serbia; 9 Sudan University of Science and Technology, Khartoum, Sudan

**Keywords:** Photovoltaic panel, Evaporative cooling, Groundwater, Electrical efficiency, Photovoltaics, Civil engineering, Mechanical engineering

## Abstract

Photovoltaic panels are considered a vital sustainable source of electrical energy; however, their efficiency tends to decline as a result of increasing temperature. This study aims to demonstrate the effectiveness of a novel evaporative cooling and groundwater-based system designed to simultaneously cool both the air and photovoltaic panels in hot, dry climates. Experimental results from the developed prototype indicate a clear enhancement in energy generation compared to conventional photovoltaic and evaporative cooling systems. Statistically, the evaporative cooling system reduced the average panel temperature by 15 °C, resulting in an 8.4% increase in photovoltaic efficiency, while maintaining air conditions of 32.6 °C and 62% relative humidity. Furthermore, the groundwater-to-air heat exchanger reduced the panel temperature by 22.8 °C, leading to a 12.7% increase in efficiency, and lowered the air temperature from 43.5 to 26.3 °C at 55% relative humidity. These results highlight the potential of integrated cooling systems to significantly improve the performance of photovoltaic panels in arid regions.

## Introduction

Solar energy is the most abundant renewable energy source on Earth, and photovoltaic (PV) power dominates in this area^1,2^. PV systems are a fundamental technology for directly converting sunlight into electrical power, and they are easy to install on building rooftops for domestic use or on large surface areas in power stations for industrial applications^3^. However, they still suffer from low conversion efficiencies, typically below 20%^4^. Moreover, prior research has shown that for every 1 °C rise in PV panel temperature beyond the standard test condition of 25 °C, electrical efficiency decreases by approximately 0.3–0.5%^5,6^. To address this issue, various cooling methods have been applied to PV panels, including both active and passive strategies, such as water cooling, air cooling, and evaporative cooling. Elminshawy et al.^7^ integrated an earth-to-air heat exchanger (EAHE) with a PV panel, achieving cooling by directing precooled ambient air to the back side of the panel. The ambient air was drawn into the buried EAHE using an air blower, then routed through cooling channels installed on the panel’s rear side. As a result, the panel temperature decreased by 8 to 13 °C, and the power output improved by 4.54% to 18.9%. Ruoping et al.^8^ coupled a PV panel with ground heat exchangers (GHEs), using circulating water to transfer heat from the panel to the ground. This configuration reduced the panel temperature by 26.8% and improved system efficiency by approximately 4.1% to 11.1%. Yang et al.^9^ developed a cooling system utilising shallow geothermal energy. Cooled water was sprayed directly onto the panel’s backside, and the runoff was collected in a basin beneath the panel. A U-shaped borehole heat exchanger (UBHE) was fixed in an existed well, allowing water to circulate between the basin and UBHE to transfer the heat groundwater. Kadhim and Aljubury^10^ suggested burying a water tank groundwater to maintain a low water temperature, which was used to spray a cooling pad mounted on the rear side of PV panel. A wide number scenarios were examined, such as spraying water over the front surface of the panel and intermittent spraying. The panel temperature was decreased by 4 °C to 12.6 °C, besides improving the efficacy from 1.74 to 16%. A cellulose pad at the bottom surface of the PV panel was introduced by Suresh and Shanmadhi^11^ implemented. A pipe with multiple water inlet holes was used to wet the pad. This configuration resulted in an average increase in electric power output of 6.8%. Almuwailhi and Zeitoun^12^ investigated both free (natural) and forced evaporative cooling of PV panels. Free evaporative cooling was achieved by fixing a PV panel above an isolated metal channel containing a wetted fabric, whereas forced evaporative cooling employed several small fans to drive air through the channel. Using free and forced evaporative cooling, the electrical efficiency of the PV panel was improved by 2.7% and 3.8%, respectively. Another investigation of a PV panel mounted on an open-top duct was developed by Haidar et al.^13^. Water flowed over a cloth placed on the lower side of the duct, and a fan was employed to circulate air within it. As a result, the panel temperature decreased by 10 °C, besides increasing power output by 5%. Agyekum et al.^14^ applied dual-surface cooling to a PV panel. The flowing water was used to cool the front side, while the backside was cooled using a wetted cotton wick mesh. This configuration increased the electrical efficacy by 11.9% and reduced the panel temperature by 23.5 °C. Alktranee and Bencs^15^ delved into passive evaporative cooling utilising cotton wicks immersed in water and attached to the backside of the PV panel. The panel temperature was decreased by approximately 22%, and the efficiency increased by 7.25%. Jafari^16^ integrated a PV panel with a geothermal heat exchanger. A coolant fluid was circulated through a mini-channel fixed to the backside of the panel and the buried geothermal exchanger. The system absorbed heat from the PV panel and rejected it into the ground, resulting in a 9.8% improvement in output power. Elghamry and Hassan^17^ embedded a PV panel into a solar chimney used to cool and ventilate a building room. The chimney was integrated with an air-tube geothermal heat exchanger. Abed et al.^18^ used a groundwater heat exchanger to assist an evaporative solar cooling system designed to cool indoor space. The system reduced the indoor air temperature by 5 °C, while relative humidity (RH) increased by 15%. The groundwater heat exchanger alone reduced indoor air temperature by up to 12 °C below the outdoor level, and RH increased to 26%. Chaichan et al.^19^ circulated groundwater through a copper coil fixed to the backside of the PV panel. This reduced temperature of the panel by 11.7 °C and improved efficiency by 16.7%. Fernandes et al.^20^ fixed a heat exchanger on the rear side of the PV panel and circulated groundwater through it to cool the panel, resulting in up to a 20% increase in power output. The previous studies presented above indicate that precooled air has been used directly to cool PV panels, despite the well-known low cooling capacity of air. Groundwater has also been utilized, for example, to enhance evaporative cooling systems for greenhouses^21,22^. Mahdi and Aljubury^23^ used groundwater directly to cool PV panels by spraying it onto their front surface. Rashid and Aljubury^24^ experimentally investigated the use of groundwater in an evaporative cooling system for a tent. Six PV panels installed on the tent’s roof powered the system. The internal air temperature in the cooled tent was reduced by 12.4 to 14.8 °C compared to the unmodified tent.

Referring to the studies discussed above, it can be concluded that the feasibility of using a combined cooling and groundwater system to simultaneously cool air and photovoltaic panels has not yet been thoroughly investigated. Therefore, this research aims to fill this gap by introducing a novel hybrid photovoltaic evaporative cooling (PV/EC) system integrated with a groundwater-to-air heat exchanger (PV/EC-GAHE).

The integrated system enables the reuse of cooled air produced during the evaporative cooling process for domestic applications. Specifically, the proposed system utilises groundwater cold water to support the PV/EC hybrid setup, in which a heat exchanger employs groundwater as a cooling coil. This heat exchanger is installed at the inlet of the evaporative cooling channel to precool the hot ambient air before it enters the EC channel.

## Materials and methods

### General description

**Fig. 1:**
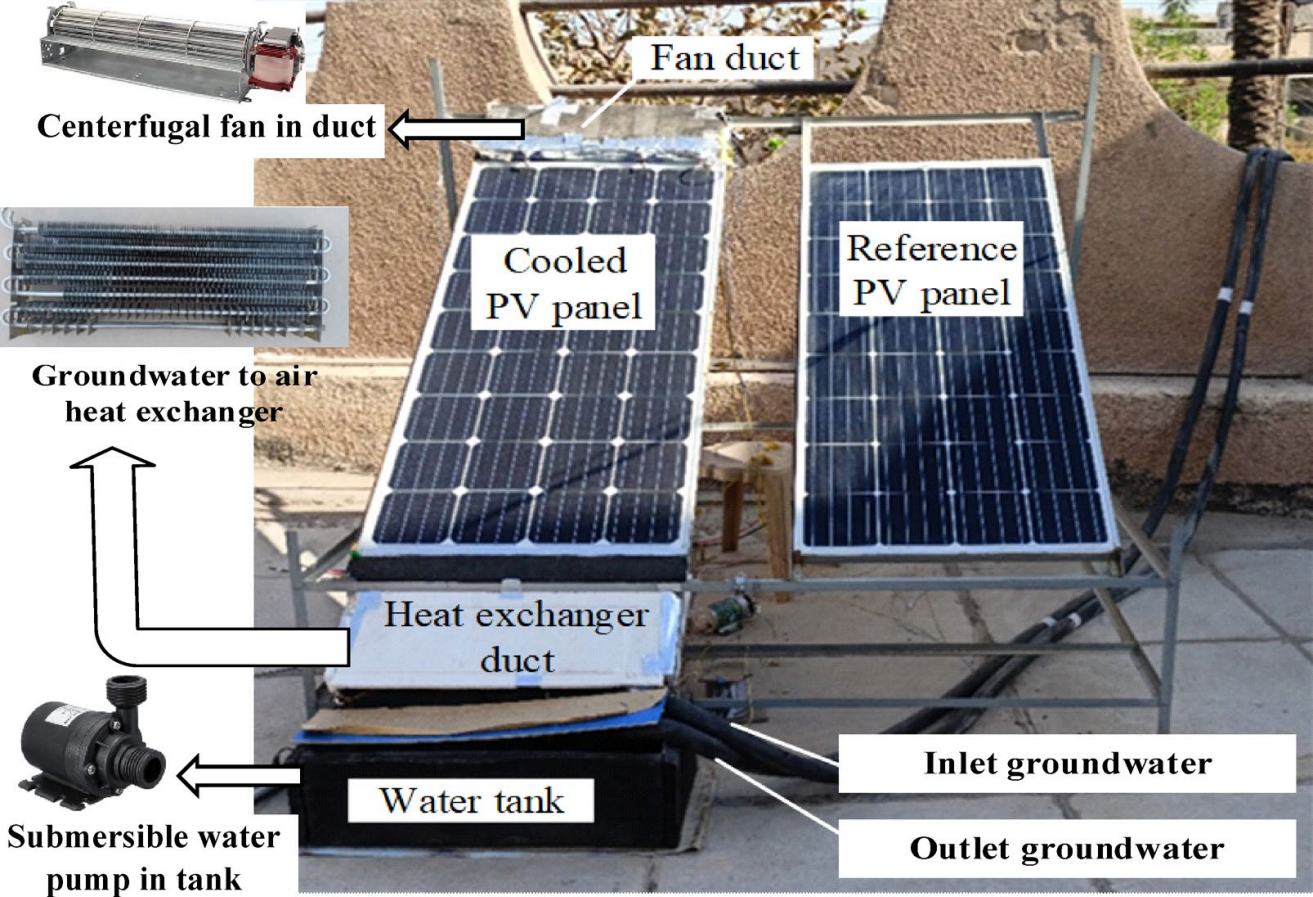
Experimental setup with groundwater-to-air heat exchanger.

The experimental test apparatus is shown in Fig. 1. It comprises two photovoltaic (PV) panels, with their specifications provided in Table 1. One of the PV panels was actively cooled, while the other panel remained uncooled and served as a reference to evaluate the effect of cooling on PV performance under identical operating conditions. To measure the surface temperature of the PV panels, five thermocouples were mounted on the back of each panel. In Baghdad (33.260118° N, 44.309133° E), the PV panels were fixed on the roof of a residential house at a height of 3 m and a tilt angle of 33.2° facing south, which corresponds to the optimal annual fixed tilt angle for Baghdad based on its latitude^25^. The solar irradiation was calculated using a TES-1333R power solar meter. The the maximum power point (Pm) and the electrical PV panel parameter open-circuit voltage (VOC) were estimated using the Solar Panel Power Multimeter (Elejoy WS-400A), which includes an internal electronic load that automatically tracks the maximum power point (Auto-MPP mode), eliminating the need for an external load.

**Table 1:** Photovoltaic panel Specification.

Brand name	SAKO
Model name	MONO-150W
Maximum power	150 W
Dimensions	1482 mm × 670 mm × 35 mm
Solar cells number	36
V_oc_	22.5 V
I_sc_	8.71 A
I_mp_	8.11 A
V_mp_	18.5 V
Weight	11.7 kg

A cellulose pad was affixed to the back-side of the cooled PV panel and positioned above an insulated metal channel. Three pad thicknesses were tested (50, 100, and 150 mm), and the 100 mm thickness demonstrated the highest performance. Therefore, the 100 mm pad was selected to be used in the current study.

**Fig. 2:**
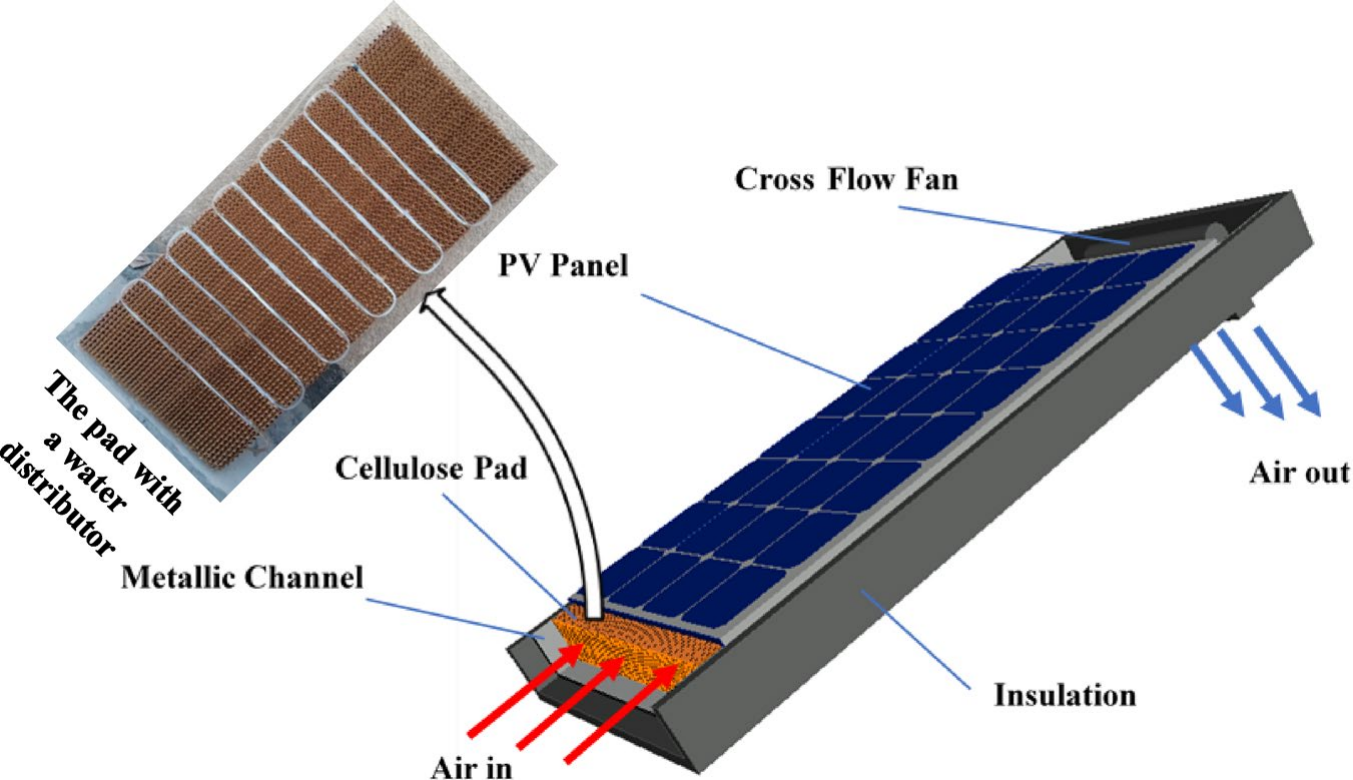
Configuration diagram of the photovoltaic evaporative cooling system.

Ambient air entered the evaporative cooling channel from the lower side, passed via the wetted cellulose pad, and exited through the upper side with the aid of an axial centrifugal fan installed at the top of the channel, which has maximum power consumed of 35 W. Additionally, a serpentine-shaped, perforated, flexible polyethylene tube was used to wet the pad and ensure uniform water distribution, as illustrated in Fig. 2. An insulated basin situated beneath the cooling channel collected the discharged water from the pad. A compact water pump (19 W) was installed inside the basin to recirculate the collected water back to the distributor tube and subsequently to the cellulose pad.

### Groundwater

A water well with a 10 cm diameter and 8 m depth was drilled. A centrifugal water pump with power of 100 W was used to extract water from the well. A novel method for developing the PV/EC system involves using cold groundwater to assist with evaporative cooling on the back-side of the PV panels by incorporating an air–water heat exchanger to pre-cool ambient air before it enters the evaporative cooling channel. The heat exchanger was placed inside an insulated air duct installed at the entrance of the evaporative cooling channel, as shown in Fig. 1. Ambient air passes through the air side of the heat exchanger, flowing over the tubes before entering the evaporative cooling channel. Simultaneously, groundwater flows via the tubes of the heat exchanger and is then discharged to a drain. To record the inlet and outlet temperatures of the groundwater, twin K-type thermo-couples were installed at the entry and exit ports of the heat exchanger. The air temperature was measured using one thermo-couple at the inlet and two thermocouples at the outlet of the heat exchanger.

### Testing procedure

**Fig. 3:**
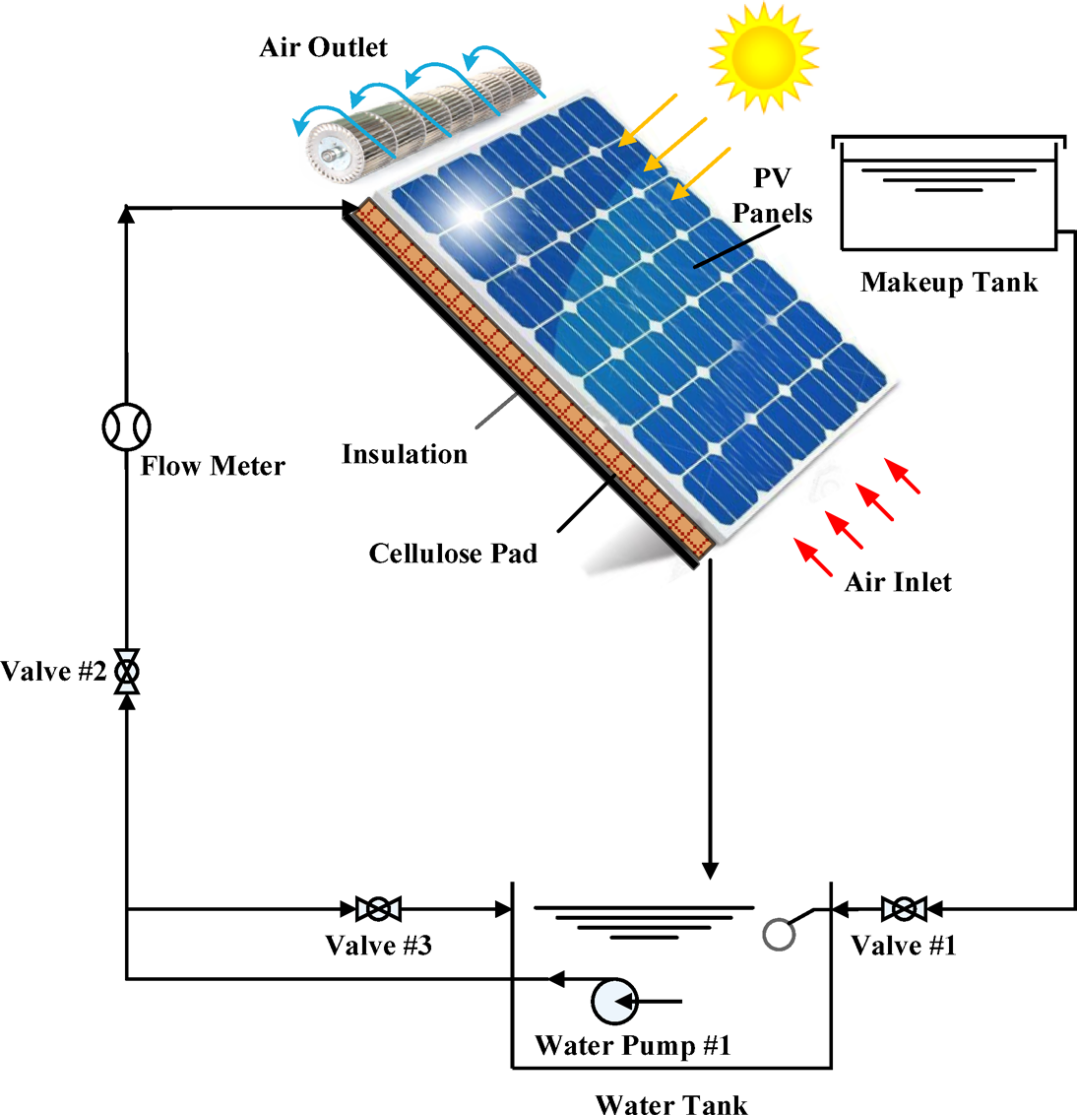
Experimental setup of PV/EC system.

Figure 3 shows the schematic diagram of the experimental setup. Experiments were conducted in August between 9:00 and 15:00 on selected sunny days. The tested period was selected to ensure full solar exposure on all photovoltaic panels, avoiding partial shading observed outside this period due to nearby structures. Thus, August was chosen for its high solar irradiance, elevated temperatures, and clear skies, ensuring stable testing conditions. The experimental procedure can be summarised in the following; initially, valve #1 was opened to fill the water basin with tap water. Water was then circulated via the evaporative cooling system utilising prime water pump #1, which pumped water from the basin to the water distribution tube to wet the cellulose pad. The drained water of the pad was returned to the basin, while evaporated water was replenished by a make-up water tank. Valves #2 and #3 were utilised to manage the water flow rate at 3 L/min. Specifically, this value was selected after testing three different water flowrates 1, 2, and 3 L/min and it was found that 3 L/min can provide the best performance.

A centrifugal cross-flow fan was employed to draw in outside air and force it via the wetted cellulose pad. Three air velocities were tested including 2, 2.5, and 3 m/s and specifically 3.0 m/s was selected for the current study due to its superior performance.

**Fig. 4:**
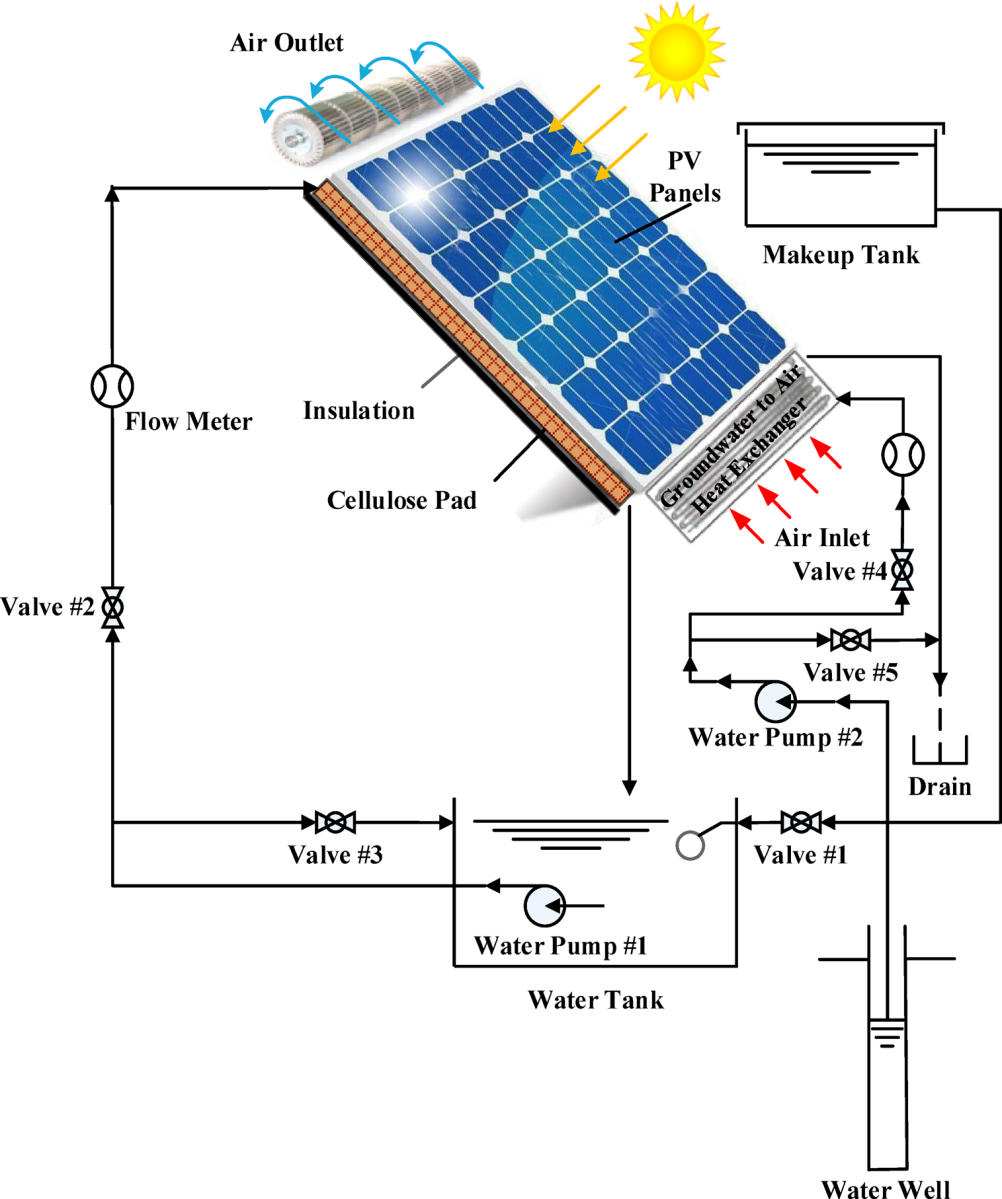
A schematic representation of photovoltaic evaporative cooling system with groundwater to air heat exchanger.

In the modified PV/EC-GAHE system, ambient air first passed through the groundwater-to-air heat exchanger (GAHE) to be precooled before entering the wetted pad, as illustrated in Fig. 4. Water pump #2 was used to circulate groundwater through the water side of the GAHE. Valves #4 and #5 were used to control the groundwater flow rate, also set to 3 L/min. Electric power, voltage, current, humidity and solar radiation were specified twice per hour, while temperatures were recorded every minute.

## Results and discussion

### Temperature variations

**Fig. 5:**
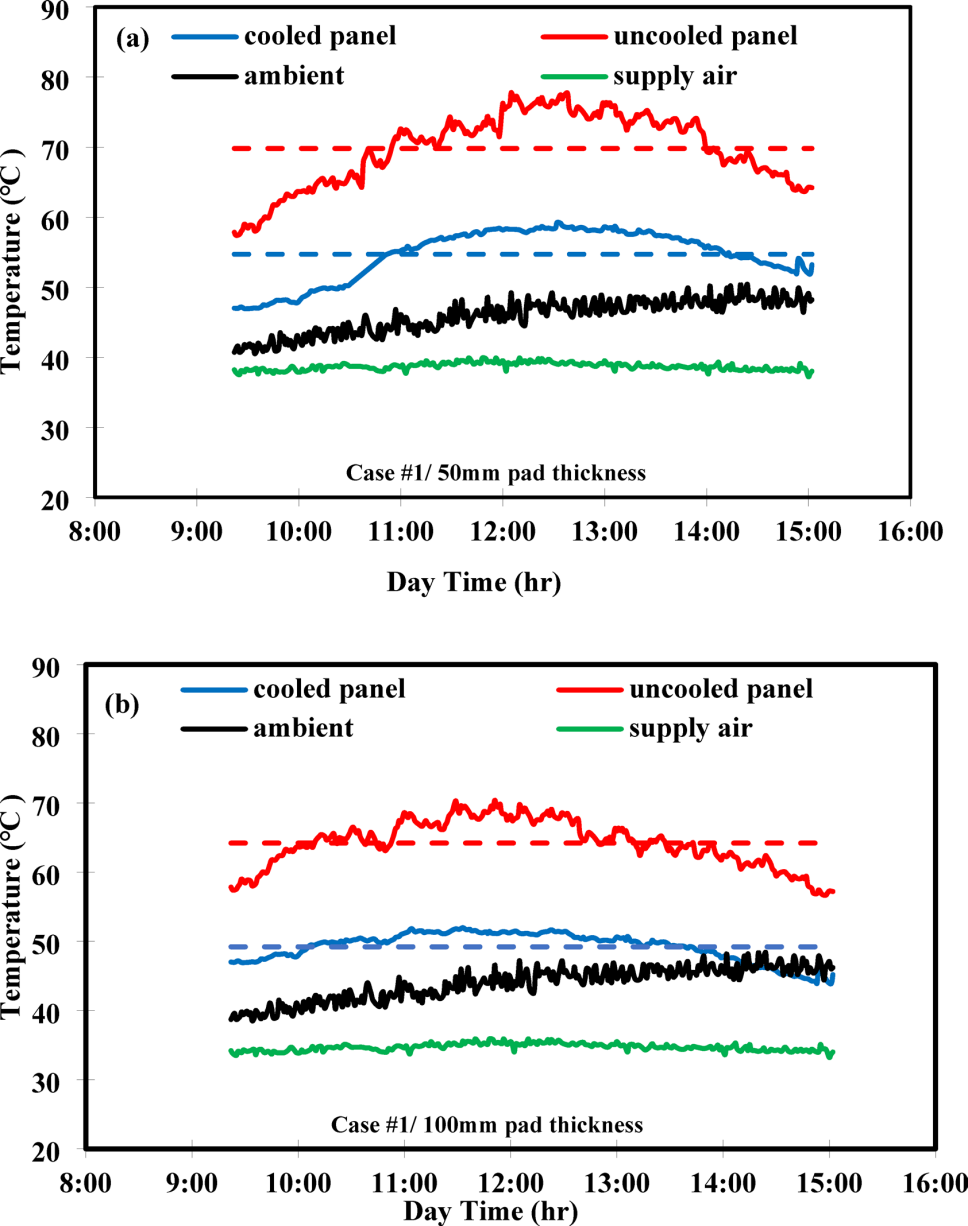

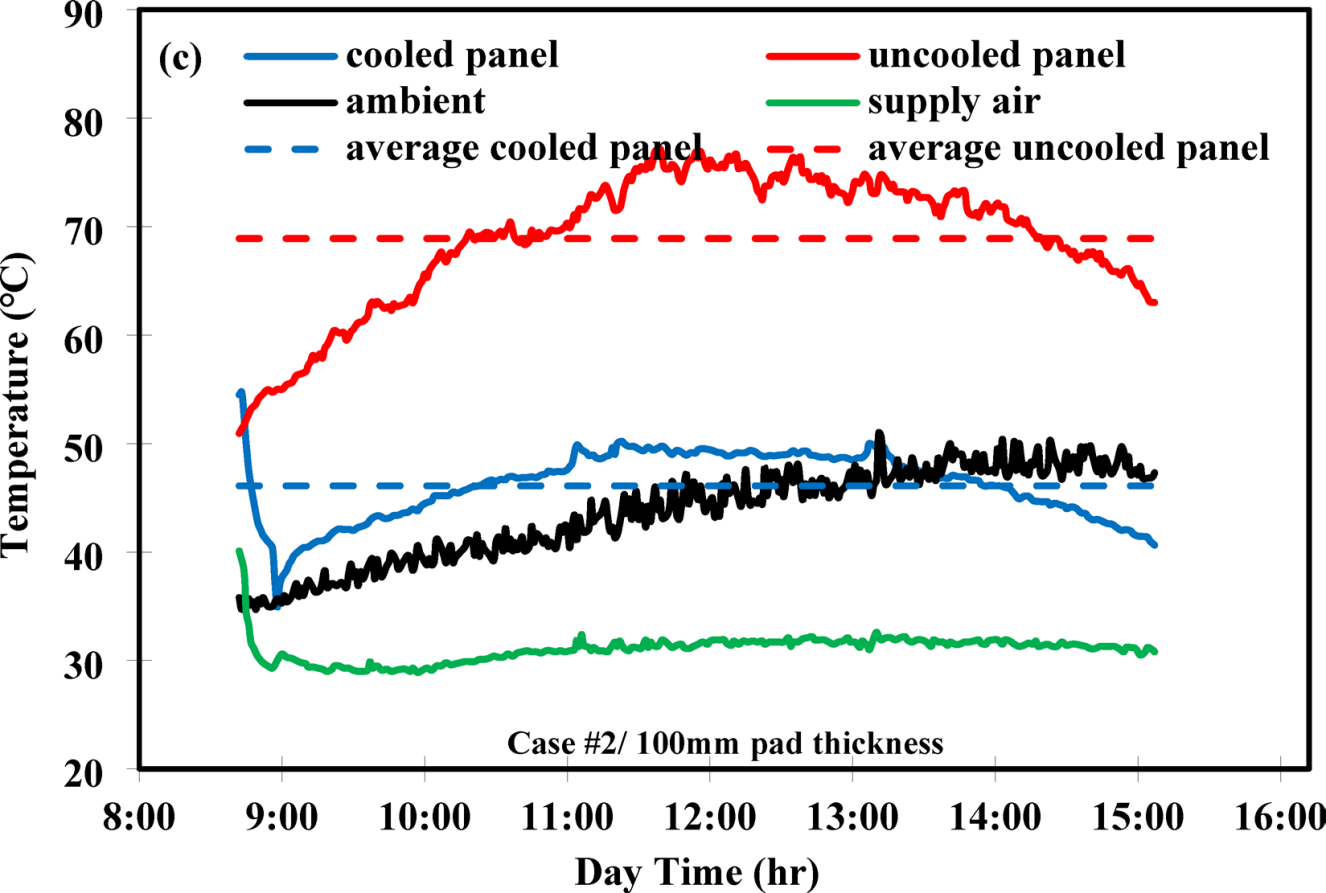
Variation of temperature against day time for (**a**) PV/EC with 50 mm pad thickness (**b**) PV/EC with 100 mm pad thickness, (**c**) PV/EC-GAHE with 100 mm, considering cooled panel, ambient, uncooled panel, supply air, average cooled and uncooled panel temperature.

The variation in temperature throughout the day for both uncooled and cooled PV panels is shown in Fig. 5. The red curve represents the temperature of the uncooled PV panels, while the blue curve depicts the temperature of the cooled panels. The red curve increases gradually, reaching its peak around noon before declining. The blue curve exhibits a similar trend but with a smaller temperature range. The average temperatures of the uncooled and cooled PV panels are indicated by red and blue dashed lines, respectively. The black curve shows the ambient air temperature, that gradually increases from 9:00 a.m. to 3:00 p.m. within the test period. The supply air temperature is represented by the green curve, which shows only slight variation over the course of the test. A slight variation in the uncooled panel temperature was observed between Case #1 (64.2 °C) and Case #2 (68.9 °C), although the tests were conducted on consecquance days under similar conditions. This minor difference can be attributed to natural fluctuation in ambient conditions between days. However, within each case, the cooled and uncooled panels were tested simultaneously and exposed to identical environmental conditions.

### Effect of the back-side evaporative cooling

Figure 5a shows the variation of temperature during the day for a 50 mm pad thickness. The uncooled PV panel’s temperature progressively raised to a maximum of 77 °C before decreasing to 64 °C. The cooled PV panel exhibited a similar trend, reaching a peak temperature of 59 °C at noon and reducing to 52 °C thereafter. This performance can be ascribed to a number of influential parameters, most notably solar radiation, which increases steadily until it peaks at noon and then declines. It was also observed that the uncooled PV panel’s temperature notably fluctuated, likely due to environmental influences, whereas the cooled panel’s temperature remained more stable due to the effect of evaporative cooling. The average temperatures of the uncooled and cooled PV panels were 69.8 °C and 54.7 °C, respectively. In this aspect, the ambient air temperature gradually rasied throughout the test period, ranging between 40 and 48 °C. The supply air temperature rose slightly, reaching 37.7 °C at noon before dropping to 34.5 °C by 3:00 p.m. Figure 5b illustrates the temperature variation for a 100 mm pad thickness. The temperature profiles followed similar patterns to those observed with the 50 mm pad. The average and maximum temperatures of the uncooled PV panel were 64.2 °C and 70.4 °C, respectively. In contrast, the cooled PV panel’s temperature did not exceed 52 °C, with a mean value of 49.2 °C. On average, there was a 15 °C temperature difference between the cooled and uncooled PV panels. The supply air temperature remained nearly constant at 34 °C, while the outside air temperature varied between 37 and 46 °C.

### Effect of groundwater

Figure 5c shows the temperature fluctuation over time when using a groundwater-cooled coil coupled with backside evaporative cooling, with 100 mm of the pad thickness. Initially, the temperatures of both the uncooled PV panel and the supplied air were relatively high but dropped sharply due to the activation of the cooling system, reaching a stable state around 9:00 AM. This behavior is not observed in the other tested cases, as measurements in those cases began only after the system had already reached stable conditions. The temperature of the uncooled PV panel rose to 76 °C at noon and then gradually decreased. In contrast, the cooled panel maintained a temperature range between 34.9  and 54.7 °C. The average temperature of the exit-cooled air was 31 °C, while the ambient air temperature ranged between 34.7  and 51 °C.

### Comparison of evaporative cooling and modification

**Fig. 6:**
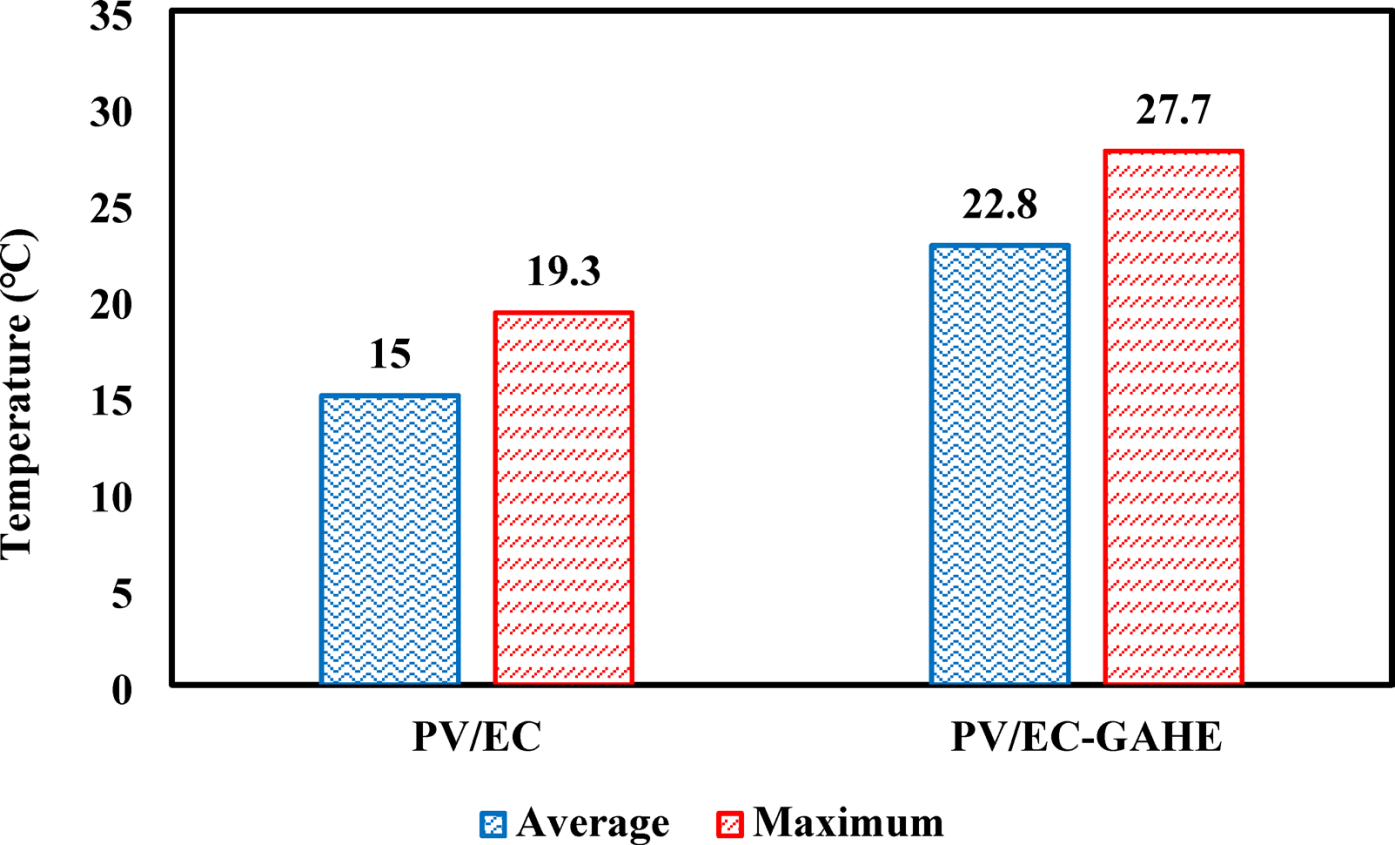
Comparison of average and maximum PV panel temperature.

The EC technique and its modified version, incorporating a GAHE, were experimentally investigated. The comparison demonstrated a significant drop in the cooled PV panel’s temperature in both cases. Both systems were tested under identical conditions, using a cooling pad thickness of 100 mm, a water flow rate of 3 L/min, and an air flow rate of 0.045 m^3^/s (corresponding to a supplied air velocity of 3 m/s). Figure 6 presents the average and maximum temperature reductions of the cooled PV panel relative to the uncooled panel for both the EC and modified systems. For the PV/EC system, the average temperature reduction was 15 °C. In the PV/EC-GAHE system, the temperature dropped by 22.8 °C, as the evaporative cooling was enhanced by precooled ambient air, achieved through the use of groundwater in the GAHE prior to entering the evaporative cooling channel. The maximum temperature drop for the PV/EC system was 19.3 °C. In contrast, the PV/EC-GAHE system achieved the greatest reduction, with the cooled PV panel reaching a maximum temperature reduction of 27.7 °C. In should be mentiond that the maximum temperature reduction of 27.7 °C was obtained after conducting a set of three experiment. Comparing to the uncooled panel temperature (76.8 °C), this would elucidate a total temperature reduction rate of 36%.

### PV panel performance

To evaluate the effect of EC and the modified system, key PV panel performance parameters, panel efficiency, power output, and open-circuit voltage, were measured. The experiments were conducted under similar operating conditions of air and water flow rates, and a cooling pad thickness of 100 mm, which was found to provide the maximum performance improvement. Additionally, the experiments were carried out on consecutive days to ensure comparable ambient temperature and solar radiation conditions across the different cooling configurations. For each case, two PV panels were simultaneously tested, one cooled and the other uncooled, to enable direct comparison. The main experimental results are summarised in Table 2.

**Table 2:** Results of the current experimental tests.

Case No	Case #1	Case #2
	PV/EC	PV/EC-GAHE
Pad thickness (mm)	100	100
Groundwater flow rate (LPM)	N/A	3
Water flow rate of the cooling pad (LPM)	3	3
Air velocity (m/s)	3	3
Uncooled panel temperature (°C)	64.2	68.9
Cooled panel temperature (°C)	49.2	46.1
Panel temperature reduction (°C)	15	22.8
Inlet air temperature (°C)	43.9	43.5
Outlet air temperature (°C)	32.6	26.3
Air temperature drop (°C)	11.3	17.2
The average power difference (W)	8.3	10.8
Maxime power difference (W)	10	12.7
Average efficiency enhancement (%)	8.4	12.7
Maximum efficiency enhancement (%)	9.3	14.9
V_oc_ for uncooled panel (V)	20.6	20.8
V_oc_ for cooled panel (V)	19.5	19.1
Enhancement of V_oc_ (%)	5.6	8.9

#### Power output

**Fig. 7:**
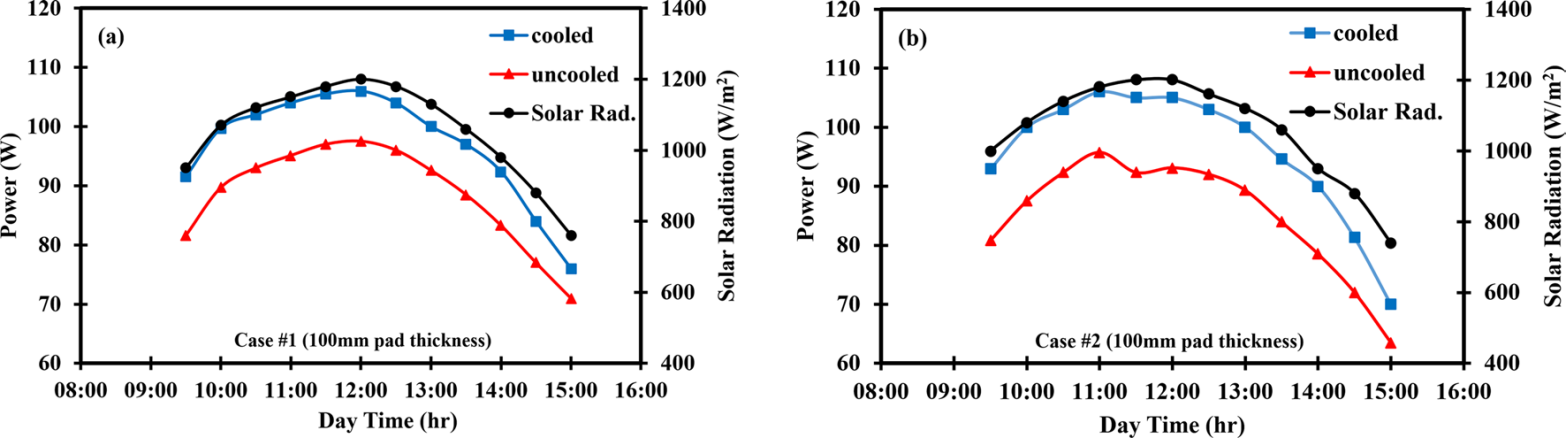
Deviation of solar radiation and power output against operational time for (**a**) PV/EC, (**b**) PV/EC-UWAH.

Figure 7 depicts the change of PV panel electric power output and solar radiation over time. It is evident that both the power output and solar radiation curves display similar behavior, progressively increasing, with a peak around midday, and then decreasing. This indicates a strong correlation between solar radiation and PV power output. Figure 7a illustrates the power output of both cooled and uncooled PV panels over operational time for the PV/EC system. The power output of the cooled panel gradually enlarged, getting a peak value of 106 W at 12:00 p.m. The average power output of the cooled and uncooled panels was 96.8 W and 88.5 W, respectively, at 1055 W/m^2^ of an average solar radiation. In the case of PV/EC-GAHE (Case #2), the use of the GAHE further enhanced the electric power output, with a maximum improvement of 12.7 W, as shown in Fig. 7b.

Referring to the calculation of energy consumption, it was revealed that the auxiliary components of PV system including the fan, pump#1 and pump#2 consume approximately 150 W and the PV panel supplies around 110 W. However, referring to case#1 that uses only one pump, the overall energy consumption would be 54 W, which can be covered by the PV panel. Thus, it should be noted that the power gain of 12.7 W made by using the case #2 of using groundwater and evaporative cooling of PV panel (Table 2) cannot cover the energy consumption of 150 W, and therefore, the two pumps were powered using the grid. Despite the net electrical gain is negative in the present small-scale prototype, this result is significantly affected by the system size, where the energy consumed by auxiliary components establishes a comparatively high fraction of the total power. Utilising the large scale PV systems, this issue can be resolved where the relative effect of these loads can be significantly lowered, and the system has the ability to attain a positive net energy balance. Furthermore, the optimal design of the proposed system can be achieved including the use of efficient pumps and fans to further lessen the energy consumption and improve the overall energy efficiency, even in small-size PV system.

#### PV panel efficiency

The electrical efficiency of the PV panel ($$\eta$$) is well-defined as the ratio of the electrical power output ($$P$$) (measured by W), to the instance solar radiation ($$G)$$ (measured by W/m^2^) on the panel and can be calculated as follows^26^:

1 $$\eta =\frac{P}{G\times A}$$

A is the front surface area of the PV panel (m^2^). Equation (2) is utilised to measure the percentage enhancement in efficiency of PV panel ($\Delta \eta$)^27^:

2 $$\Delta \eta =\frac{{\eta }_{c}-{\eta }_{ref}}{{\eta }_{ref}}\times 100\%$$

η_c_ η_ref_ signify the efficiency of the cooled and uncooled PV panels.

**Fig. 8:**
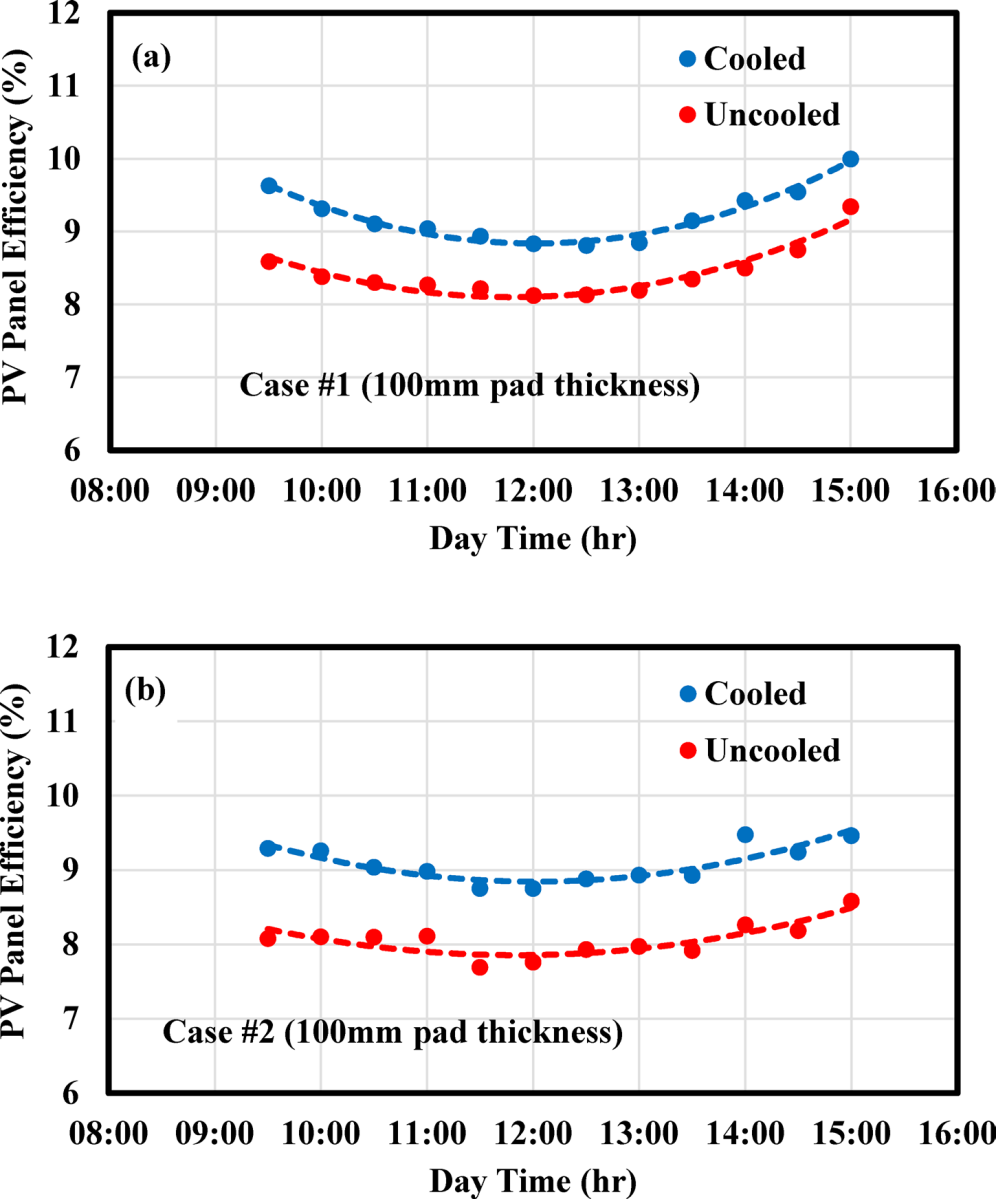
Discrepancy of PV panel efficiency against operational time for (**a**) PV/EC, (**b**) PV/EC-GAHE.

The efficiency of the cooled and uncooled PV panels varies over time, as shown in Fig. 8. All the curves exhibit a similar pattern: efficiency decreases gradually, reaching a minimum around noon, and then increases steadily in the afternoon. This trend can be explained by the fact that PV efficiency is inversely proportional to solar radiation and openly relative to power output and. Since solar radiation varies significantly throughout the day, particularly peaking at noon, while power output varies less drastically, the resulting efficiency curve reflects this imbalance. Additionally, panel temperature, like solar radiation, peaks around noon, which further contributes to the drop in efficiency due to the negative effect of temperature on PV performance. Figure 8a presents the variation in efficiency for the uncooled and cooled PV panels in Case #1. It can be observed that the enhancement in efficiency remains nearly constant over time, with a maximum increase of approximately 9.3%. In Case #2, as shown in Fig. 8b, the cooled PV panel consistently outperformed the uncooled panel, achieving a maximum efficiency enhancement of 14.9%. Specifically, this value was obtained after achieving a set of three experiments, where the average value was considered.

#### Open circuit voltage

**Fig. 9:**
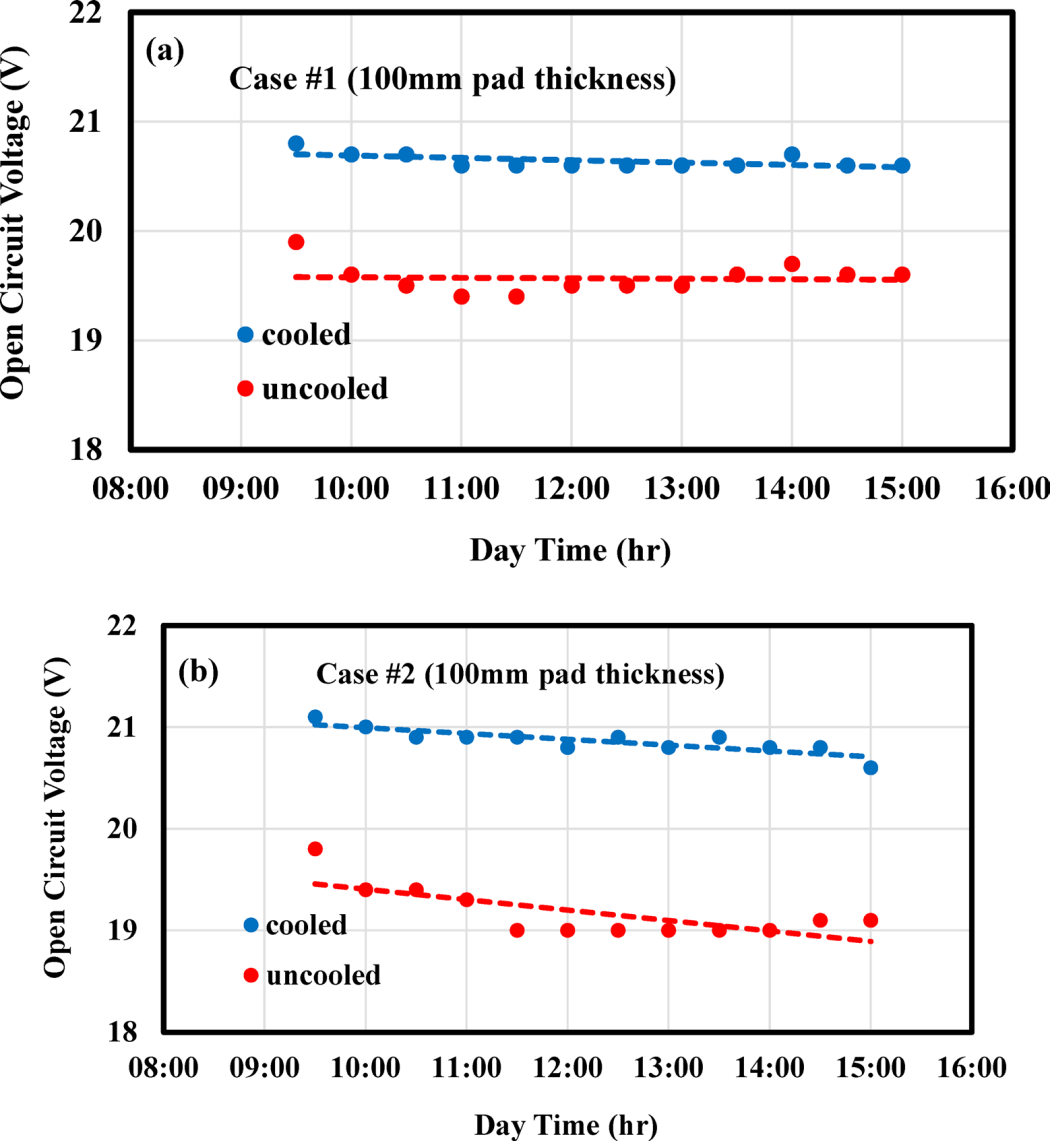
Discrepancy of open circuit voltage of PV panel against operational time for (**a**) PV/EC, (**b**) PV/EC-GAHE.

The open-circuit voltage (V_OC_) is an imperative factor for evaluating the performance of a PV panel and is significantly impacted by temperature. As the PV panel temperature increases, the voltage typically decreases. Figure 9 shows the variation of V_OC_ over the course of the day for both cooled and uncooled panels. In all cases, V_OC_ varied linearly with time, and the voltage of the cooled panel was consistently higher than that of the uncooled panel. In Fig. 9a, the V_OC_ of both the cooled and uncooled panels remained nearly constant throughout the test period, with the cooled panel showing a 5.6% higher voltage than the uncooled one. In Case #2, where groundwater cooling was employed, the V_OC_ increased by approximately 8.9%, as shown in Fig. 9b. It should be noted that the increase of 8.9%, in V_OC_ remains within the manufacturer’s nominal voltage range under standard test conditions (STC), which is 21.6 V. Therefore, the stability of the solar panel for a long-term operation would not be significantly affected.

Referring to the experimental results of Table 2, the dual application of cooled air from the hybrid evaporative and groundwater cooling system can be identified. Specifically, the developed technique has enhanced the overall efficiency of photovoltaic panels besides contributing to indoor comfort in arid climates. By attaining a noticeable 17.2 °C reduction in air temperature, the developed system van provide a more pleasant and conducive environment for occupants, predominantly in regions where extreme heat can obstruct daily activities. Indeed, the cooling effect has improved indoor air quality by maintaining optimal relative humidity levels. This integrated technique of using cooled air for both energy generation and human comfort can highlight the multifaceted advantages of the developed system, delivering a sustainable solution that discourses both energy competence and livability in hot, dry regions.

### Uncertainty analysis

#### Temperature uncertainty

Each experiment involves some errors that must be identified. The uncertainty analysis discussed here is intended to ensure the previous sections’ results are accurate and are not caused by errors. The uncertainty was carried over to the temperature measurements since it is one of the significant variable in this study. The total error (*E*_*t*_) is computed as follows^28^:

3 $${E}_{t}=\sqrt{{E}_{P}^{2}+{E}_{B}^{2}}$$

Referring to the manufacturer, the bias error (*E*_*B*_), which is equal to 1 °C. Table 3 signifies the measurements of eight temperature from the same thermo-couple. *Ep* denotes the calculated precision error using the following measurements of Table 3.

**Table 3:** Temperature measurements for calculating (*Ep*).

N	1	2	3	4	5	6	7	8
T (°C)	50.1	50.1	50.3	50.1	50.2	50.3	50.1	50.1

4 $${E}_{p}=\frac{SD}{\sqrt{n}}{t}_{v,\frac{\varphi }{2}}$$

Referring to Eq. (4), n is the number of readings, SD is the standard deviation, t_v, φ/2_ is the t-distribution at the degree of freedom v = n − 1, with a confidence level of 95%. Then φ/2 = 0.025, and from the t-distribution table, t7, 0.025 = 2.365, and the precision error *E*_*p*_ = 0.0766°C, then the total error *E*_*t*_ = 1.0029 °C. Since the difference in temperature during the experiments is more accurate than the total error. The experimental results can be regarded as reliable and accurate.

#### Uncertainty of PV panel efficiency

The precision of the influential variables including I, V and G has a considerable impact on the uncertainty of the electrical efficiency of PV panel ($\upeta )$. Therefore, the uncertainty $(\omega \eta )$ of the demonstrated efficiency can be attributed to to uncertainties in discrete variable,which can be estimated utilising the least-square fit analysis as demonstrated by Holman (2011) and stated below:

5 $$\omega R={\left[{\left(\frac{\partial R}{\partial {x}_{1}}{\omega }_{1}\right)}^{2}+{\left(\frac{\partial R}{\partial {x}_{2}}{\omega }_{2}\right)}^{2}+\dots +{\left(\frac{\partial R}{\partial {x}_{n}}{\omega }_{n}\right)}^{2}\right]}^\frac{1}{2}$$

6 $$\upeta =\frac{{V}_{m} {I}_{m}}{\text{G A}}$$

7 $$\omega \eta ={\left[{\left(\frac{\partial \eta }{\partial {V}_{m}}{\omega }_{{V}_{m}}\right)}^{2}+{\left(\frac{\partial \eta }{\partial {I}_{m}}{\omega }_{{I}_{m}}\right)}^{2}+{\left(\frac{\partial \eta }{\partial G}{\omega }_{G}\right)}^{2}\right]}^\frac{1}{2}$$

8 $$\frac{\partial \eta }{\partial {V}_{m}}=\frac{{I}_{m}}{\text{G}\times \text{A}}$$

9 $$\frac{\partial \eta }{\partial {I}_{m}}=\frac{{V}_{m}}{\text{G A}}$$

10 $$\frac{\partial \eta }{\partial G}=\frac{-{V}_{m} {I}_{m}}{{\text{G}}^{2}\text{ A}}$$

The uncertainty of the PV panel efficiency is 0.201%.

## Conclusions and recommendations for future research

A novel hybrid photovoltaic evaporative cooling system was developed and enhanced using groundwater. A cellulose pad was directly connected to the rear side of the PV panel, with dry ambient air and recirculating cold water flowing over the pad to facilitate evaporative cooling. Additionally, groundwater was utilised by integrating a water-to-air heat exchanger at the inlet of the evaporative cooling duct, pre-cooling the ambient air before it entered the system. This innovative hybrid system simultaneously cooled the PV panel, generated electrical energy, and supplied cold, humid air suitable for thermal comfort.

The key findings of this study are summarized as follows:

- The average and maximum reductions in PV panel temperature due to evaporative cooling alone (with a 100 mm pad thickness) were 15 °C and 19.3 °C, respectively.
- Integrating a groundwater-to-air heat exchanger (GAHE) with the PV/EC hybrid system (PV/EC-GAHE) reduced the ambient air temperature entering the evaporative channel by 10 °C, resulting in a maximum PV panel temperature drop of 27.7 °C.
- Evaporative cooling improved the average electrical efficiency of the PV panel by 8.4%, while the use of GAHE with EC further increased the average efficiency to 12.7%.
- The PV panel temperature decreased by an average of 15 °C with EC alone and 22.8 °C with the GAHE system, corresponding to increases in power output of 8.3 W and 10.8 W, respectively.
- The open-circuit voltage (VOC) was enhanced by 5.6% without GAHE and by 8.9% with GAHE.
- The supply air temperature was reduced from 43.5 to 26.3 °C (a reduction of 17.2 °C) at 55% relative humidity through the application of GAHE, reaching the thermal comfort zone. This indicates that the evaporative cooling system maintains favorable and sustainable conditions, ensuring efficient cooling and preventing excessive moisture buildup, which supports reliable long-term system performance.
- It is fair to expect that the efficiency gains obtained may be negatively impacted by adverse environmental conditions such as dust storms and water scarcity.

For future research, it is recommended to:

- Evaluate the performance of the proposed system at full scale to assess its feasibility and efficiency under real operating conditions.
- Investigate various configurations of the groundwater-to-air heat exchanger, including tube diameter, number of rows, and arrangement, to optimise thermal performance.
- Conduct a numerical study of the groundwater-to-air heat exchanger to simulate its thermal behavior and support further design enhancements.
- Development of an adaptive control systems capable of adjusting water and air flow in real time based on solar irradiance and ambient temperature is important, with the goal of maximising cooling performance and energy efficiency.

## Data Availability

The data supporting the findings of this study can be accessed from corresponding author upon reasonable request.

## List of symbols

A: Area, m^2^
E_B_: Bias error
E_p_: Precision error
E_t_: Total error
G: Solar radiation, W/m^2^
I_mp_: Current maximum power, A
I_sc_: Short circuit current, A
n: Number of measurements
P: Electric power, W
P_m_: Maximum power, W
SD: Standard deviation
T: Temperature, ℃
v: Degree of freedom
V_mp_: Voltage of maximum power, V
V_OC_: Open circuit voltage, V
Δη: Percentage enhancement efficiency, %
η: PV panel electrical efficiency, %

## Abbreviations

EC: Evaporative cooling
PV: Photovoltaic
GAHE: Groundwater-to-air heat exchanger

## Subscripts

c: Cooled PV panel
ref: Reference PV panel (uncooled panel)

## References

[1] Hussein, A. K. et al. A review of the application of hybrid nanofluids in solar still energy systems and guidelines for future prospects. *Sol. Energy* **272**, 112485 (2024).

[2] Khariy, A. H., Ajel, A. R., Gharghan, S. K. & Noaman, N. M. Grid-connected PV system for 324 kW with improved maximum powerpoint tracking. *J. Tech.* **5**(2), 20–31 (2023).

[3] Mohammad, A., Al-Obaidi, M., Dakkama, H. & Bahlol, H. Modelling and optimisation of solar photovoltaic power using response surface methodology. *J. Sustain. Dev. Energy Water Environ. Syst.* **12**(4), 1–16 (2024).

[4] Zanlorenzi, G., Szejka, A. L. & Canciglieri, O. Hybrid photovoltaic module for efficiency improvement through an automatic water cooling system: A prototype case study. *J. Clean. Prod.* **196**, 535–546. 10.1016/j.jclepro.2018.06.065 (2018).

[5] Haidar, Z. A., Orfi, J., Oztop, H. F. & Kaneesamkandi, Z. Cooling of solar PV panels using evaporative cooling. *J. Therm. Eng.* **2**(5), 928–933. 10.18186/jte.72554 (2016).

[6] Kadhim, A. M. & Aljubury, I. M. A. Experimental evaluation of evaporative cooling for enhancing photovoltaic panels efficiency using underground water. *J. Eng.* **26**(8), 14–33. 10.31026/j.eng.2020.08.02 (2020).

[7] Elminshawy, N. A. S., Mohamed, A. M. I., Morad, K., Elhenawy, Y. & Alrobaian, A. A. Performance of PV panel coupled with geothermal air cooling system subjected to hot climatic. *Appl. Therm. Eng.* **148**, 1–9. 10.1016/j.applthermaleng.2018.11.027 (2019).

[8] Ruoping, Y., Xiaohui, Y., Fuwei, L. & Huajun, W. Study of operation performance for a solar photovoltaic system assisted cooling by ground heat exchangers in arid climate, China. *Renew. Energy* **155**, 102–110. 10.1016/j.renene.2020.03.109 (2020).

[9] Yang, L. H., De Liang, J., Hsu, C. Y., Yang, T. H. & Chen, S. L. Enhanced efficiency of photovoltaic panels by integrating a spray cooling system with shallow geothermal energy heat exchanger. *Renew. Energy* **134**, 970–981. 10.1016/j.renene.2018.11.089 (2019).

[10] Kadhim, A. M. & Aljubury, I. M. A. Experimental performance of cooling photovoltaic panels using geothermal energy in an arid climate. *Heat Transf.* **50**(3), 2725–2742. 10.1002/htj.22002 (2021).

[11] Suresh, M. & Shanmadhi, R. Studies on the performance of 150W solar photovoltaic module with evaporative cooling. *IOP Conf. Ser. Mater. Sci. Eng.* **912**, 4. 10.1088/1757-899X/912/4/042016 (2020).

[12] Almuwailhi, A. & Zeitoun, O. Investigating the cooling of solar photovoltaic modules under the conditions of Riyadh. *J. King Saud Univ. Eng. Sci.*10.1016/j.jksues.2021.03.007 (2021).

[13] Haidar, Z. A., Orfi, J. & Kaneesamkandi, Z. Photovoltaic panels temperature regulation using evaporative cooling principle: Detailed theoretical and real operating conditions experimental approaches. *Energies* **14**, 1. 10.3390/en14010145 (2021).

[14] Agyekum, E. B., PraveenKumar, S., Alwan, N. T., Velkin, V. I. & Shcheklein, S. E. Effect of dual surface cooling of solar photovoltaic panel on the efficiency of the module: Experimental investigation. *Heliyon* **7**(9), e07920. 10.1016/j.heliyon.2021.e07920 (2021). 34522812 10.1016/j.heliyon.2021.e07920 PMC8424511

[15] Alktranee, M. & Bencs, P. Effect of evaporative cooling on photovoltaic module performance. *Process. Integr. Optim. Sustain.*10.1007/s41660-022-00268-w (2022).

[16] Jafari, R. Optimization and energy analysis of a novel geothermal heat exchanger for photovoltaic panel cooling. *Sol. Energy* **226**, 122–133. 10.1016/j.solener.2021.08.046 (2021).

[17] Elghamry, R. & Hassan, H. Impact a combination of geothermal and solar energy systems on building ventilation, heating and output power: Experimental study. *Renew. Energy* **152**, 1403–1413. 10.1016/j.renene.2020.01.107 (2020).

[18] Abed, F. M., Zaidan, M. H., Hasanuzzaman, M., Kumar, L. & Jasim, A. K. Modelling and experimental performance investigation of a transpired solar collector and underground heat exchanger assisted hybrid evaporative cooling system. *J. Build. Eng.* **44**, 102620. 10.1016/j.jobe.2021.102620 (2021).

[19] Chaichan, M. T. et al. Assessment cooling of photovoltaic modules using underground water. *Arab. Gulf J. Sci. Res.* **39**(2), 151–169. 10.51758/agjsr-02-2021-0016 (2021).

[20] Fernandes, F. T., Farret, F. A., Longo, A. J., de Nardin, C. R. & Trapp, J. G. PV efficiency improvement by underground heat exchanging and heat storage. In *3rd Renewable Power Generation Conference (RPG 2014)* 1–6 (2014). 10.1049/cp.2014.0875

[21] Aljubury, I. M. A. & Ridha, H. D. A. Enhancement of evaporative cooling system in a greenhouse using geothermal energy. *Renew. Energy* **111**, 321–331. 10.1016/j.renene.2017.03.080 (2017).

[22] Alhosainy, A. H. & Aljubury, I. M. A. Two stage evaporative cooling of residential building using geothermal energy. *J. Eng.* **25**(4), 29–44 (2019).

[23] Mahdi, A. H. & Ali Aljubury, I. M. Experimental investigation of two-stage evaporative cooler powered by photovoltaic panels using underground water. *J. Build. Eng.* **44**, 102679. 10.1016/j.jobe.2021.102679 (2021).

[24] Rashid, M. A. & Ali Aljubury, I. M. Impact of two stage evaporative cooling system using underground water powered by PV panels on improving the thermal comfort of emergency relief tents in dry-hot climate regions. *Energy Build.* **310**, 114100 (2024).

[25] Bailek, N. et al. Optimized fixed tilt for incident solar energy maximization on flat surfaces located in the Algerian Big South. *Sustain. Energy Technol. Assess.* **28**, 96–102. 10.1016/j.seta.2018.06.002 (2018).

[26] Sainthiya, H., Beniwal, N. S. & Garg, N. Efficiency improvement of a photovoltaic module using front surface cooling method in summer and winter conditions. *J. Sol. Energy Eng. Trans. ASME* **140**(6), 1–7. 10.1115/1.4040238 (2018).

[27] Haidar, Z. A., Orfi, J. & Kaneesamkandi, Z. Experimental investigation of evaporative cooling for enhancing photovoltaic panels efficiency. *Results Phys.* **11**, 690–697. 10.1016/j.rinp.2018.10.016 (2018).

[28] Thomas, G. B., Roy, D. M. & John, H. L. *Mechanical Measurements*, 6th ed. (2011).

